# Identification of the contribution of contact and aerial biomechanical parameters in acrobatic performance

**DOI:** 10.1371/journal.pone.0172083

**Published:** 2017-04-19

**Authors:** Diane Haering, Aurore Huchez, Franck Barbier, Patrice Holvoët, Mickaël Begon

**Affiliations:** 1 Institut National de Recherche en Informatique et Automatique Rennes - Bretagne Atlantique, Campus de Beaulieu, Rennes, France; 2 Université de Montréal, Department of Kinesiology, Laboratory of Simulation & Movement Modeling, Laval, Québec, Canada; 3 Université de Valenciennes et du Hainaut Cambrésis, Laboratoire d’Automatique et Mécanique Industrielles et Humaines, Le Mont Houy, Valenciennes, France; 4 Université de Lille 2, Faculté des Sciences du Sport et de l’Education Physique, Ronchin, France; INSEP, FRANCE

## Abstract

**Introduction:**

Teaching acrobatic skills with a minimal amount of repetition is a major challenge for coaches. Biomechanical, statistical or computer simulation tools can help them identify the most determinant factors of performance. Release parameters, change in moment of inertia and segmental momentum transfers were identified in the prediction of acrobatics success. The purpose of the present study was to evaluate the relative contribution of these parameters in performance throughout expertise or optimisation based improvements. The counter movement forward in flight (CMFIF) was chosen for its intrinsic dichotomy between the accessibility of its attempt and complexity of its mastery.

**Methods:**

Three repetitions of the CMFIF performed by eight novice and eight advanced female gymnasts were recorded using a motion capture system. Optimal aerial techniques that maximise rotation potential at regrasp were also computed. A 14-segment-multibody-model defined through the Rigid Body Dynamics Library was used to compute recorded and optimal kinematics, and biomechanical parameters. A stepwise multiple linear regression was used to determine the relative contribution of these parameters in novice recorded, novice optimised, advanced recorded and advanced optimised trials. Finally, fixed effects of expertise and optimisation were tested through a mixed-effects analysis.

**Results and discussion:**

Variation in release state only contributed to performances in novice recorded trials. Moment of inertia contribution to performance increased from novice recorded, to novice optimised, advanced recorded, and advanced optimised trials. Contribution to performance of momentum transfer to the trunk during the flight prevailed in all recorded trials. Although optimisation decreased transfer contribution, momentum transfer to the arms appeared.

**Conclusion:**

Findings suggest that novices should be coached on both contact and aerial technique. Inversely, mainly improved aerial technique helped advanced gymnasts increase their performance. For both, reduction of the moment of inertia should be focused on. The method proposed in this article could be generalized to any aerial skill learning investigation.

## Introduction

In acrobatic sports, the main objective is to master movements with rotations performed during an aerial phase. Most injuries are due to the repetitive nature of their learning [1]. Hence, minimizing the number of repetitions has become a major challenge for coaches [2]. In regards to recent work, this could be achieved by helping coaches to focus their observation and feedback on the most relevant cues using knowledge-based shortcuts [3, 4]. Biomechanical tools can indeed be used to identify the most determining performance factors based on either statistics [5, 6] or computer simulation models [7].

**Fig 1:**
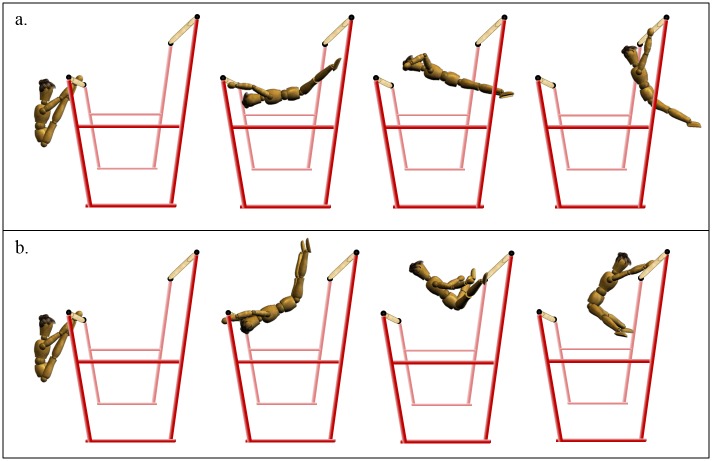
Three-dimensional representation of the counter movement forward in flight. (a) Novice performance. (b) Advanced performance.

Investigation in acrobatics learning has mostly relied on comparisons between different levels of expertise [8, 9]. Optimization has also been used to compare recorded *versus* optimal performances [10, 11]. The combination of expertise and optimization comparisons can identify rooms for improvement for both novices and more advanced gymnasts [12]. In this prospect, the counter movement forward in flight (CMFIF) is a transition move on the uneven bars described by the *Fédération Internationale de Gymnastique* as an “underswing on the low bar [with feet support] with counter movement forward in flight to hang high bar” [13] that can challenge gymnasts of varying expertise. Typically, novices are likely to perform it although their body rotation at re-grasp is insufficient to swing and perform an upstart in sequence (Fig 1). Expert gymnasts also commonly perform the skill, but they rarely manage it without deduction in competition. In fact, while it is performed in approximately 70% of the routines, only 30% of them meet the exigence of the judges [14]. Therefore, this skill seems relevant to investigate performance improvement by means of comparison between groups of varying expertise or optimization. More precisely, deductions apply after regrasp if the shoulder level is lower than the upper bar (0.3 point), the feet are passed the vertical position of the shoulders (0.1 point), or limited swing leads to a lack of rhythm in the following element (0.1 point) or an additional swing (0.5 point, FIG code of points, 2014; p. 51–52). Therefore, experts are expected to produce larger vertical and rotational components to avoid such deductions. The performance definition should be related to the potential of the gymnast to effectively link the skill.

The trajectory and rotation potential of the athlete during the aerial phase of the skill are the result of a contact phase, during which linear and angular momenta are generated. Coordination and strength are required to produce large momenta during a short contact duration and large linear velocities toward the best direction for height and distance [15, 16]. Studies on tumbling, vault or bars showed that more expert gymnasts exhibited larger vertical velocities and shorter contact times, and suggested that appropriate take-off conditions are the most important success predicators [17–20]. According to these results, focusing the learning on contact phase technique to produce better release parameters appears to be a good strategy. However, the control of body rotation through the aerial phase technique is another complex task that could influence the final performance of acrobatics skills. In fact, body rotation can be controlled by moment of inertia adjustments and by angular momentum transfers between segments. Such adjustments are mainly related to the technique and motor control based on visual and proprioceptive information [21, 22]. Indeed in high bar dismounts, elite gymnasts were able to minimize their moment of inertia in a larger extend than non-elite gymnasts by means of hip and knee flexion [23]. It has also been evidenced that segmental contribution to the angular or linear momentum can differ according to expertise level or between subjects of the same performance level. In particular in skills that require a reversal of rotation direction similar to the CMFIF, Brüggemann, Cheetham (1994) [24] highlighted the importance of leg contribution.

Besides, skill difficulty can also be influenced by landing or regrasp complexity. The movement is thus constrained at the same time by the desired aerial rotation and the landing or regrasp conditions. Therefore, all phases of acrobatic movements (contact, aerial, and landing or regrasp phases) are interrelated. The first two phases affect the success of the subsequent while the last two phases constrain the mechanics of the previous ones. In fact, both release state (i.e. the initial rotation, linear and angular velocities of the aerial phase) and aerial joint kinematics were found to differ to various extents between advanced and novices in acrobatics [12, 25], In the meantime within each phase, parameters interact. For example, transfer of angular momentum is affected by changes in the moment of inertia in between phases or during a same phase [26]. Nevertheless, no study identified which has the largest influence on final performance. In that way, Pijnappels, Kingma (2010) [27] quantified by a numerical calculation the contribution of arm movements to balance. The analysis of relative contribution of release state, variation of inertia moment, and individual segmental rotation in advanced *versus* novice performances could help direct learning towards more effective methods [28].

Computer simulation coupled with multiple linear regression are interesting tools to estimate those contributions. First, simulation is achieved through model generation that simplifies the calculation of movement biomechanical parameters. Second, take-off state and aerial joint kinematics optimisation is performed [17, 29] to identify possibilities for acrobatics improvement and guide coaches toward technical or physical cues for either novice or expert gymnasts improvement. Third, multiple linear regression allows mathematical calculation for independent parameters contribution to the performance [30].

Despite many studies investigating either contact release state or changes in the moment of inertia or angular momentum transfer as parameters influencing acrobatic performances, none focused on the relative contribution of each of these parameters to performance. Therefore, the main objective of this study was to assess contribution of biomechanical parameters to acrobatic performance in accordance with the expertise level for recorded and maximal (obtained by dynamic optimisation) performances. The difficulty for the CMFIF relies mostly on the continuation of the movement after re-grasping rather than the possibility to re-grasp itself. In a previous study, linear velocity norm and transversal angular momentum at release were found to be similar between novice and expert gymnasts [20]. Therefore, we hypothesized that improvement of the performance in novice and advanced gymnasts mainly occurs through aerial joint kinematics adjustments to control the rotation of the body rather than to modify release state to improve the body trajectory toward the upper bar. However, a secondary hypothesis is that optimization of aerial kinematics is not sufficient for novice gymnasts to reach advanced performance level.

## Methods

### Experimental protocol

Experiment of the study was approved by the Ethic Committee of the University of Valenciennes. Written consent was also obtained from the participants and from their parents for participants under 18. The uneven bars were setup in line with the competition rules [31]. All gymnasts performed three trials of CMFIFs in sequence with an upstart if possible. The kinematics of 35 markers placed on gymnasts and 4 markers locating uneven bars were recorded during all trials using a motion analysis system (10 Vicon T20 cameras @250 Hz). Three-dimensional joint kinematics of a 14-segment multibody system (i.e. trunk, head, arm, forearm, hand, thigh, shank and foot of left and right sides) were computed following ISB recommendations [32, 33]. Since the markers were placed to be visible throughout the movement, some corrections were applied to determine the anatomical axes of flexion, abduction and rotation (S1 File).

In order to form two groups of different level with remaining room for improvement, five novice (20.5±2.3 years, 1.66±0.07 m, 56.1±6.6 kg) female gymnasts able to attempt CMFIF and six national-level (13.7±2.9 years, 1.51±0.08 m, 42.5±10.1 kg) female gymnasts able to link the CMFIF with a kip to support but with no previous performance of it in competition were selected. A performance score was calculated as the horizontal coordinate of the CoM at re-grasp (time *t* = *T*) with respect to the high bar: $G_{H}^{T}$ [12]. This CoM position relative to high bar corresponds to the moment arm of the body weight calculated around the bar in the sagittal plane to initiate the backward swing after an aerial forward rotation. Specifically, based on their average performance score of the CMFIF, seven advanced gymnasts ($G_{Hadv}^{T}=-0.36\pm 0.10$) and seven novices gymnasts ($G_{Hnov}^{T}=-0.08\pm 0.08$) were discriminated in groups statistically different (p<0.001).

### Simulation

To generate the optimal aerial techniques for each advanced and novice gymnasts, a parametric angle driven model was created through the Rigid Body Dynamics Library [34] based on our previous model [12]. The model was personalized according to an anthropometrical model and marker-based anatomical centers of rotation [35].

For simulation purpose, knee, ankle, neck and wrist joint angles were fixed; movement of the trunk was assumed planar; and upper and lower limbs were actuated symmetrically throughout aerial phase. As the root segment, the trunk had three DoFs, **q**_**1**_, (forward and vertical translations, and forward rotation) in the spatial reference frame associated to high bar. The hip flexion and abduction, the shoulder plane of elevation, forward elevation and axial rotation, and the elbow flexion and prono-supination were seven driven DoFs, **q**_**2**_. Based on the Euler-Lagrange equation without contact, the acceleration of the root segment ${\ddot{\text{q}}}_{1}$ was a function of the multibody system state $x\left(t\right)={\left[{\text{q}}_{1}^{t}\;{\text{q}}_{2}^{t}\;{\dot{\text{q}}}_{1}^{t}\;{\dot{\text{q}}}_{2}^{t}\right]}^{\text{T}}$ and the joint accelerations $\ddot{\text{q}}2(t)$. This ordinary differential equation (ODE) was solved using a 4-5^th^ order Runge-Kutta algorithm from the release state ***x***^0^ to the final time, which corresponded to an event equation in the ODE solver. This event was defined as either the gymnast catching the high bar or her wrists passing beyond the high bar vertical plane.

### Optimisation

For the two groups of gymnasts, optimal in-flight kinematics were computed from recorded release state parameters as in Huchez, et al. (2015) [12] with additional constraints about the shoulder kinematics. In summary, the joint angle time histories during the CMFIF of the model were fitted by quartic splines [36] defined by 36 parameters consisting in four nodes plus one time-derivative at the last node for the seven joint angles of the model, plus the total time.

The spline parameters were optimized to obtain in-flight kinematics that maximizes the performance criterion defined as the horizontal coordinate of the centre of mass relative to the high bar at the final state: $J=\underset{\left[X,t\right]}{\text{max}G_{H}^{T}}$, under nonlinear constraints. The optimized kinematics and the time derivative of the final state were bounded with respect to the maximal values measured in the 42 actual trials.

**Fig 2:**
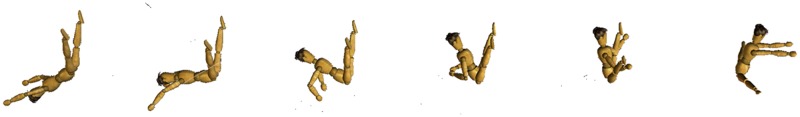
Example of an unrealistic arm kinematics found in optimal solution using our previous model [12].

The nonlinear constraints were: successful re-grasp defined by wrist back under the bar and finger joint in front above it; hand mediolateral axis collinear (±40°) to the bar; and hand to hand distance between 0.2 and 0.6 m [12]. For enhanced realism, crossing between the legs and the lower-arms or high bar were avoided using a line-cylinder intersection algorithm where legs were line segments and lower-arms or bar were 3 or 2 cm radius cylinder respectively. The main improvement in the model with respect to our previous computer simulation model was the implementation of a shoulder range of motion constraint that accounts for degrees-of-freedom interactions [37] in order to avoid non-realistic solutions obtained in previous work (Fig 2). The method used for this implementation is described in S1 File.

### Data reduction and statistics

Four conditions arose from the combination of expertise (novice *vs* advanced) and the optimisation (actual *vs* optimal). For all trials, performance scores ($G_{H}^{T}$) were obtained. In the meanwhile, components that relate to performance by means of controlling global body and segments rotations were identified. On one hand, the global body rotation included the rotation due to the release state (*θ*_*RS*_) and the additional rotation due to the change in moment of inertia (*θ*_*ΔI*_). On the other hand, the segments rotations relative to the global body are defined by complementary rotations of legs (*θ*_*TRlegs*_), arms (*θ*_*TRarms*_) and trunk (*θ*_*TRtrunk*_) thanks to segmental angular momentum transfers. These components were calculated using angular velocity of the body at release (*ω*_*0*_ *= σ*_*0*_/*I*_*0*_), the moment of inertia time history (*I*(*t*)), and the segments angular velocity time histories (*ω*_*i*_) such as:

$$\theta_{RS}=\omega_{0}\left(t_{f}-t_{0}\right)$$ (1a)

$$\theta_{\Delta I}=\int_{t_{0}}^{t_{f}}\frac{\sigma_{0}}{I\left(t\right)}dt-\theta_{RS}$$ (1b)

$$\theta_{TRi}=\int_{t_{0}}^{t_{f}}\omega_{i}dt-\theta_{RS}-\theta_{\Delta I}$$ (1c)

Pearson correlation matrices were chosen to estimate a linear relationship between performance and rotation components in each group. Only coefficients of correlation with absolute values higher than 0.5 were considered to describe a correlation at 0.05 significance level (N = 18 or N = 24). At that point, the correlation matrix cross-products were used to verify variables non-collinearity. As numerous collinear relationships were identified between all independent variables, a stepwise multiple regression method was used to select from all rotation components only those significantly contributing to increase the performance prediction while avoiding collinearities [38]. P-values, F-values and root mean square error of the resulting multiple linear regression models were also reported to validate the models. Then, for each group a predicted performance (${\text{y}}_{G_{H}^{T}}$) based on rotation components can be obtained based on the following regression equation:

$${\text{y}}_{G_{H}^{T}}=\beta_{1}.\;\theta_{RS}+\beta_{2}.\theta_{\Delta I}+\beta_{3}.\theta_{TRlegs}+\beta_{4}.\theta_{TRarms}+\beta_{5}.\theta_{TRtrunk}+\text{c}+\varepsilon ,$$ (2a)

where β_1−5_ are each rotation component coefficient that is null when the corresponding component has no significant influence on performance, *c* is the intercept constant, and *ε* is the residual root mean square error in the model.

Finally, the relative contribution of release parameters, $x_{RS}=\left(\beta_{1}.\theta_{\text{RS}}\right)/({\text{y}}_{G_{H}^{T}}-c)$, variation in moment of inertia, $x_{\Delta I}=\left(\beta_{2}.\theta_{\Delta \text{I}}\right)/({\text{y}}_{G_{H}^{T}}-c)$, and the sum of segmental angular momentum transfers, $x_{TR}=\left(\beta_{3}.\theta_{\text{TRlegs}}+\beta_{4}.\theta_{\text{TRarms}}+\beta_{5}.\theta_{\text{TRtrunk}}\right)/({\text{y}}_{G_{H}^{T}}-c)$, to the performance predicted by the regression model, ${\text{y}}_{G_{H}^{T}}$, were identified for each group of trials verifying:

$${\text{x}}_{\text{RS}}+{\text{x}}_{\Delta \text{I}}+{\text{x}}_{\text{TR}}=1.$$ (2b)

A linear mixed-effects analysis was computed on performance scores and relative contribution of angular momentum, moment of inertia and momentum transfers to test the effects of expertise, optimization, and the interaction between the effects of expertise and the effects of optimization investigated in the ANOVA. Since independence of the two variables was not found, the expertise*optimisation interaction is then considered as an extra variable for the statistical analysis. A linear mixed-effect method was preferred to a classical ANOVA procedure in order to include a classical independent group comparison (novice *versus* expert), a repeated measures comparison (before *versus* after optimization), and account for inter-individual or inter-trial effects at the same time with no need of normal distribution. Therefore, expertise, optimisation and expertise*optimisation interaction were defined as the fixed effects of the model while subject and trial corresponded to the random effects.

## Results

First, the rotation component in recorded trials that correlates the most with performance is additional rotation of the trunk with coefficients of r = 0.86 et r = 0.85 for novices and advanced gymnasts respectively. This correlation is smaller with optimised trials, but still exists. In second, the rotation due to moment of inertia variation is also correlated for recorded (r = 0.78, 0.65) and optimised (r = 0.50, 0.71) trials of novice and advanced gymnasts. The individual rotation of the legs also correlates with performance in both recorded and optimised trials for advanced only (r = 0.73, 0.66). Positive correlation is also found between rotation due to release state and novice recorded performance, while negative correlation is found for advanced optimised performance and isolated rotation of the arms. The correlation matrices also highlight positive correlation between rotation of the legs and moment of inertia variation or trunk rotation in advanced.

**Fig 3:**
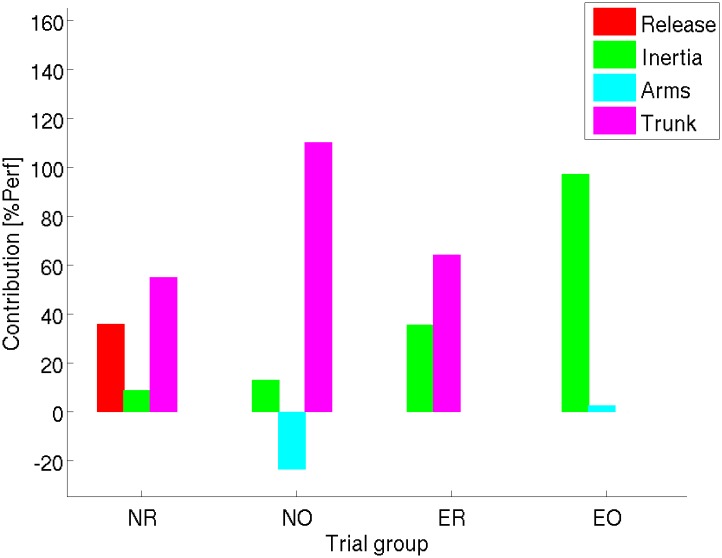
Relative contribution of biomechanical parameters to average performance of each group of trials. Release state (red), change in inertia (green), or angular momentum transfer to the legs (blue), arms (cyan) and trunk (magenta).

The stepwise multiple linear regression models for predicting performance from rotation parameters for each trial group are presented in Table 1. For all four groups, the largest coefficients are attributed to variation of inertia, while individual rotation of the legs shows no significant influence in the final model. In addition, for recorded performances, and novice optimised trials, coefficients are attributed to rotation of the trunk. Fig 3 displays the percentage each significant contribution represents to the performance. Concerning relative contribution of parameters, variation of inertia is the most important only in advanced optimised performance. For the other three conditions, rotation of the trunk contributes itself for more than 50% to the final performance. Small coefficients and contribution of the arms also becomes significant in optimised performances. Lastly, release state helps to predict novice recorded performance only. *P*-values smaller than 0.001 (and root mean square errors <0.05 cm) are found for each model indicating a very good confidence interval above 99.9%. In addition, no correlation is found between any of the selected predictive parameters in each model (Table 1). *F*-values for recorded trials are larger than for optimised trials indicating that at least one factor contributes with stronger evidence to predict performance.

**Table 1:** Multiple linear regression parameters for the independent rotation variables used to predict performance with matching p-values, F and rmse.

	c	θ_RS_	θ_ΔI_	θ_TRlegs_	θ_TRarms_	θ_TRtrunk_	p-value	F-value	*ε* [m]
novice recorded	0.28*	0.11*	0.43*	-0.03	0.02	0.27*	0.000	98.53	0.0265
novice optimised	0.02*	-0.13	0.25*	-0.02	-0.02*	0.05*	0.000	22.45	0.0496
advanced recorded	0.28*	0.13	0.74*	0.03	0.01	0.30*	0.000	82.96	0.0285
advanced optimised	-0.13*	0.02	0.53*	0.03	-0.03*	0.05	0.000	15.70	0.0430

* indicates parameters that contribute significantly (p<0.05) to increase the regression model prediction precision.

According to the mixed linear analysis, interaction between expertise and optimisation is significant to all contributions provided by the regression models (Table 2) and performance. In addition, individual fixed effect of expertise is also significant for release and moment of inertia contribution. Fixed effect of optimisation is significant for inertia and transfer contributions. Contribution of inertia variation is almost null in novice recorded trials, and increases quite constantly from novice recorded into novice optimised, expert recorded and expert optimised trials (Table 3). Conversely, contribution of segmental momentum transfer is significantly smaller in advanced or optimal performances than their counterparts. However, in regard to the expertise*optimisation interaction, only its contribution in novice recorded trials is significant and non-null.

**Table 2:** Fixed effects of expertise, optimisation and expertise*optimisation interaction on performance and contributions of biomechanical parameters.

	$y_{G_{H}^{T}}$ (m)		x_RS_ (%)		x_ΔI_ (%)		x_TR_ (%)	
Fixed effects	p	F	p	F	p	F	p	F
Expertise	<0.001	51.02	<0.001	135.24	<0.01	7.55	0.34	0.91
Optimisation	<0.001	42.22	<0.001	248.9	0.63	0.23	<0.001	12.73
Exp*Opt	0.01	6.67	<0.001	106.67	<0.001	18.16	<0.001	47.16

(Exp*Opt) expertise-optimisation interaction, ($y_{G_{H}^{T}}$) performance, (x_RS_) release state, (x_ΔI_) moment of inertia change, and (x_TR_) segmental momentum transfer.

**Table 3:** Regression model parameters for predicting performance in each group of trials.

Expertise/Optimisation	$y_{G_{H}^{T}}$ (m)	x_RS_ (%)	x_ΔI_ (%)	x_TR_ (%)
	[mean ± SD]	[mean ± SD]	[mean ± SD]	[mean ± SD]
Novice recorded	0.01±0.10	36±16	9±11	55±13
Novice optimal	0.12±0.08	0±0	13±55	87±55
Advanced recorded	0.27±0.09	0±0	36±6	64±6
Advanced optimal	0.31±0.06	0±0	97±22	3±22

Means and standard deviations of modelled performance $y_{G_{H}^{T}}$, and contributions of release state x_RS_, moment of inertia change x_ΔI_, and segmental momentum transfer x_TR_.

**Fig 4:**
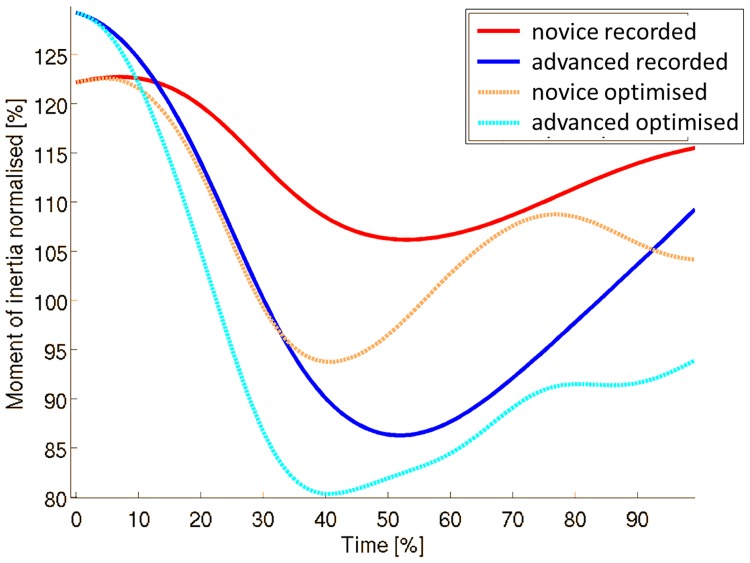
Average time-histories of the transversal moment of inertia normalised to the gymnast moment of inertia in anatomical position for each group of trials.

When looking closer at transversal moment of inertia, average initial values are larger (p = 0.003) in advanced than in novice gymnasts (Fig 4). In contrast, minimum moment of inertia attained during the flight is smaller (p = 0.000) in advanced than in novice gymnasts. Furthermore, in optimised trials, the minimum is reached earlier (p = 0.000) than in recorded performances and a more reduced (p = 0.000) moment of inertia remains at regrasp compared to recorded ones.

**Fig 5:**
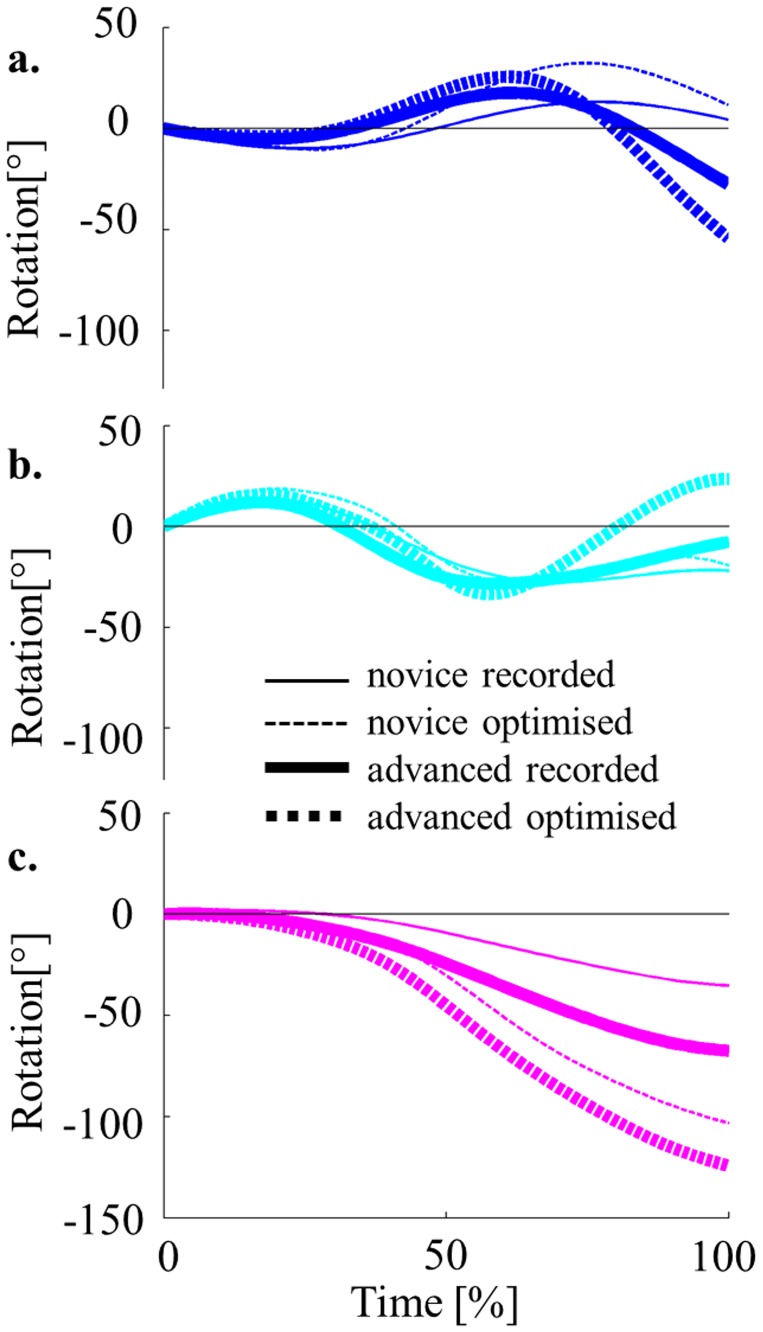
Average transversal rotation time-history for each group of trials. Legs (a.), Arms (b.), and Trunk (c.) relative to the global body (0°).

Focusing on the rotation of the legs, arms and trunk relative to the global body, main differences between groups appear in the second half of the flight (Fig 5). At regrasp, rotation of the legs relative to the global body is negative in advanced trials but positive in novices. Similarly, rotation of the arms relative to the global body is also negative in all groups, except the advanced optimal group in the second half of the flight. Trunk is the only segment which rotation relative to the global body is negative in all groups. In addition, trunk rotation to global body rotation discrepancy increases throughout the entire movement. Both expertise and optimization increase positive additional rotation of the legs relative to the global body at regrasp.

## Discussion

This study aimed at estimating the relative contribution of contact and aerial biomechanical components to the performance of the CMFIF. Overall, results suggest that performance is influenced by contact and aerial components in terms of release state, moment of inertia reduction and individual segment rotation through angular momentum transfers. However, contribution of those parameters vary with expertise and optimisation.

Among the three components of rotation related to performance, release state among trials did not statistically contribute to the level of performance in three out of four conditions. This result seems contradictory with previous studies on Tkatchev release skills that demonstrated the primary importance of linear and angular momenta for the successful performance of the skill [39, 40]. In the present study, release state seems to influence performance only in novice recorded trials, where it contributed to more than 30% of the performance. Those results might be due to the fact that the CMFIF is a low physically demanding skill that can be performed by novice gymnasts in whom release requirements might be demanding in a similar manner than Tkatchev releases were for expert gymnasts [41]. Considering the skill difficulty as well as the gymnast expertise may then be required to appropriately focus their learning. In addition, an energetic analysis of the different performances in regards to the gymnast physical condition might be of interest to investigate that question. Moreover, the release state contribution in optimised performances should be interpreted with caution since only aerial kinematics is optimised. Furthermore, with optimal in-flight joint kinematics, the novice performance remains significantly inferior to advanced actual performance. Therefore, improving release state at early stage of learning acrobatics skills remains necessary.

More precisely however, the same body rotation may be obtained with a large angular momentum and a short aerial phase (novices) or inversely (advanced). In earlier studies, coordination and power were found to be key in producing large momenta during short push-off duration or swing [15, 16, 42, 43], and large linear velocities toward the best direction for height and distance. An actual trade-off between those two elements was shown through the study of different types of back somersaults from simple (single tucked) to very difficult (double layout) by Hraski and Mejovsek (2004) [44]. In previous study, release state directly induced by contact technique displayed some significant differences between novice and advanced gymnasts in terms of hip flexion velocity and release angle but none in terms of linear velocity and angular momentum [20]. Therefore, the main parameter with novice gymnasts during the contact phase is expected to be the moment the gymnast releases the bar to reach a better release angle rather than a modification of the contact technique to modify linear and angular momentum. Thereafter, the duration of the CMFIF flight phase before re-grasping the high bar remains the highest constraint. Larger angle or vertical velocity of the body at release might be aimed at. The in-flight duration could be a key factor to change moment of inertia and momentum transfer. While optimisation in the present study was limited to aerial phase, contact phase leading to the release state might be the focus of a future study to confirm this hypothesis. Moreover, the release state contribution in optimized performances should be interpreted with caution since only aerial kinematics is optimized. Furthermore, the evolution of the skill in the women gymnastics code of point is based on variations of the contact and push off technique from pike circle with toes on the bar as studied here, unto clear hip or straddle underswing before release [13]. Then according to previous work, the optimisation of contact parameters in all those forms of swing is expected to rely upon gymnast strength and flexibility potential [45] which could then be taken into account for an optimisation of this phase.

Later on, the interest for learning might come from the aerial components of performance at any learning stage. Release parameters do not appear to have the largest contribution to performance in any group. Precedent results based on optimized advanced technique [12] suggested three combined in-flight strategies helped improve performance: 1) increase hip flexion-abduction to reduce transverse moment of inertia, 2) transfer leg and arm angular momenta to the increase forward rotation of the trunk and 3) a straighter hand path to the bar. However, results were not applied to novices and relative contribution of release state, moment of inertia variation and angular momenta transfers were not quantified. Firstly, in agreement, moment of inertia variation contributed to performance in all conditions. Its average contribution showed a significant continuous improvement from novice recorded trials, to novice optimised trials, advanced recorded trials, and finally advanced optimised trials.

In fact, transverse moment of inertia was decreased more in advanced than in novice gymnasts, starting from a slightly more stretched position (larger initial value) at release into a more piked and straddled position (smaller minimum value) in the air. Similar optimal adaptations in novices and advanced tended to decrease even more the minimum value, but mostly to adopt a small moment of inertia value for a longer period. Indeed, the legs are straddled faster and then kept in straddle position until regrasp. Increased hip extensors and adductors flexibility as well as greater flexors and abductors strength might then be required, since hip angular velocity and torques limits implemented for the optimisation were based on the study of Sheets and Hubbard (2008) [46] corresponding to an elite gymnast.

Brüggemann, Cheetham (1994) [24] found a prevalent contribution of the legs angular momentum at release in Tkatchev performances. During the flight in the present results, trunk rotation displayed the largest correlation and contribution to performance in novice and advanced recorded trials. Therefore, gymnasts seem to rely on techniques that increase angular momentum transfer from limbs to trunk to improve their performance. The role of the arms in this process was also significant for novice optimised performances. Through aerial technique optimisation, momentum transfer contribution was indeed increased in novices but became insignificant to performance in advanced for whom no additional segment rotation relative to the whole body significantly influenced performance. The absence of significance of momentum transfer contribution in advanced optimised performances does not mean that transfer does not exist, but that the contribution of inertia to reach this stage predominates. As expected, transfer and moment of inertia contribution to that point might influence each other [26] and display collinearity factors as they are both related to legs kinematics. Therefore, gymnasts might benefit from focusing on their moment of inertia reduction by piking and straddling their legs as much as they can rather than blocking their legs from rotating in order to rotate their trunk (S1a & S1c versus S1b & S1d Fig respectively), even if the result might present similarities.

Furthermore, to refine their aerial technique, it seems that optimal performance put a significant importance on the arms contribution. Even more from a motor control point of view, hand control trajectory in regrasp tasks might be important at the end of the aerial phase [47]. In addition, adopting a straighter path to the bar may allow a more favourable time-accuracy trade-off to the regrasp [48]. Therefore, focusing on a straighter path of the hand to the high bar or even rotating the arms backward or downward could help make a difference between final performances (S1c versus S1d Fig).

Finally, both novice and advanced gymnasts should focus toward improving their aerial technique. In fact, optimised trials suggest that novices could improve more from increasing momentum transfer between arms and trunk, while advanced mostly increase contribution of moment of inertia reduction. The ability of decreasing their moment of inertia is crucial to increase rotation speed throughout the aerial phase.

A significant difference was found between recorded and optimized advanced performances. Since optimal solutions respect physiological constraints based on gymnast measurement, this difference indicates that gymnasts from the present study were experts in gymnastics, but were not able to perform the CMFIF without penalties. In addition, the optimisation criterion that we chose does not take into account the constraint to deal with variability inherent to the human movement due to noise present in the environment for example [49]. Indeed, the further the gymnast centre of mass distance from the high bar when the gymnast should catch is, the smaller the room for adjustments remains. For this type of acrobatic moves, if the gymnast misses the bar, not only the performance is unsuccessful, but the consequences of a fall might be large (e.g. hands or head would hit the floor first).

The gap between optimized novice and advanced recorded performances remains as large and significant as the one filled by optimising aerial technique. Still, the contact technique refinement could possibly contribute less than aerial technique can in novice gymnasts, which does confirm our hypothesis. Nevertheless, the different contribution of contact and aerial parameters in novice and advanced or recorded and optimised trials also confirms that a non-linear coaching approach focusing on varying aspects of the skill should be recommended [50]. In addition, large variability observed in computed contributions advocates that gymnast’s individualized non-linear learning might be even more appropriate.

## Conclusion

To identify the mechanical components of body rotation is recommended to personalize acrobatics learning to specific gymnasts or skills. For the CMFIF, the contribution of each mechanical component varied between novice and advanced gymnasts, but also between recorded and optimized techniques. Findings suggest that novices should be coached on both contact and aerial technique, since their performance with optimized aerial kinematics remains lower than advanced recorded performance. Conversely, advanced gymnasts mainly increase their performance through aerial technique improvement. For both, in regards to optimized aerial solutions, enabling larger change in moment of inertia seemed the best perspective to improve their aerial technique. In addition, the method proposed in this article could be applied to other types of acrobatics skill to identify specific or general rules to help coaches.

## Supporting information

S1 File Implementation of the shoulder range of motion constraint taking into account three-dimensional degrees-of-freedom interaction. (DOCX)

S1 Fig Two-dimensional 4-segments representation of the aerial sequence of movement closest to average for each group of trial. Novice recorded(a.), novice optimal (b.), advanced recorded (c.), and advanced optimal performances (d.). Smaller lengths in the (y,z) plane means the segments are more abducted towards x axis.
(TIF)

S2 Fig Constraint cost computation steps. (a) joint limit definition and adjustment inside boundaries of a complete revolute joint, (b) discretization of the entire space and set the relative position to joint limits, (c) test of shoulder joint constraint for realistic (left) and unrealistic (right) poses.
(TIFF)
