# Supplementary material for: Identification of the contribution of contact and aerial biomechanical parameters in acrobatic performance
Source: PLoS One. 2017 Apr 19;12(4):e0172083. doi: 10.1371/journal.pone.0172083 (PMC5396868; doi:10.1371/journal.pone.0172083)
Supplement: S1 File — (DOCX) [file pone.0172083.s001.docx]

**S1 File. Implementation of the shoulder range of motion constraint taking into account three-dimensional degrees-of-freedom interaction.**

To maximize sports performances, maximal joint range-of-movement may be approached or even reached. Therefore, implementation of physiological joints limits into the models directly affects realism of simulated movements and therefore is crucial for the optimal synthesis of human movements. For revolute joints enabling movement along one degree-of-freedom, minimum and maximum range-of-motion boundaries meet the requirement. However, most joints of the body are multi-dimensional and interactions appear between ranges-of-motion of their respective degrees-of-freedom. Therefore unrealistic solutions arise from task and degrees-of-freedom kinematical redundancies [[51](#_ENREF_50), [52](#_ENREF_51)]. In the human body, mainly arm movements are sensitive to this problem, since shoulder is the most mobile and complex joint including several subsidiary joints to move the arm about three degrees-of-freedom at least [[53](#_ENREF_52)]. A common method used to define joint limits with a two degrees-of-freedom interaction are the sinus cones [[54](#_ENREF_53)] or reach-cone joint limits [[55](#_ENREF_54)]. Methods derivate from sinus cone were proposed to integrate a third degree-of-freedom, but independently from the first two [[56](#_ENREF_55)]. Recently, a quaternion field boundaries method was proposed to consider all interactions between the three degrees-of-freedom of the shoulder for animation of movements [[57](#_ENREF_56)]. However, this method has not been adapted for dynamics optimization yet and would make results difficult to interpret. Shoulder range-of-motion limits accounting for three degrees-of-freedom interactions based on Euler angles calculation were also defined [[58](#_ENREF_57)].

Implementation of these limits into a simulation model efficiently helped discriminate realistic from unrealistic poses [[59](#_ENREF_58)]. Because such an implementation within a dynamic optimization algorithm should also consider computing efficiency [[60](#_ENREF_59)], proposed a RoM constraint parametrization method consisting in space discretization associated to Boolean values. This method was yet applied to joint reach-cone limits. Its application to Euler angles shoulder limits would be of interest for constraining arm movement during gymnastics movement optimisation.

First a three-dimensional range of motion accounting for degrees-of-freedom interaction was defined to match the simulation model degrees of freedom definition. Therefore, following the method described in [Haering, Raison (2014) [58]](#_ENREF_57), three-dimensional (3D) angular space of movement of the arm defined by the ZXZ Euler sequence was computed. Gymnasts are usually more flexible at the shoulder than average population [[61-63](#_ENREF_60)]. Therefore, positions reached by all the subjects were included to define the maximal angular space. In this case, normalization procedure to obtain average volume was unnecessary [[58](#_ENREF_57)]. To ensure that actual performances of the CMFIF are included into the angular space. The latter was enlarged of a 10° tolerance (S2 Fig a).

**S2 Fig. Constraint cost computation steps.** (a) joint limit definition and adjustment inside boundaries of a complete revolute joint, (b) discretization of the entire space and set the relative position to joint limits, (c) test of shoulder joint constraint for realistic (left) and unrealistic (right) poses.

For the optimization, a non-linear constraint was implemented by discretizing the angular space into cubes with sides of 1° lengths (S2 Fig b). Each arm pose tested by the optimisation was then attributed the shortest distance between the corresponding cube and the 3D joint limits hull (S2 Fig c). If the tested pose is inside joint limit, the returned constraint is respected (negative); whereas if it falls outside the limits, the constraint is not respected (positive). Joint torques was also bounded based on a female gymnast isokinetic measurement [[64](#_ENREF_63)] to ensure realistic joint accelerations. A mutli-start optimisation algorithm as in [Huchez, Haering (2015) [12]](#_ENREF_12), was used to test the effect of the constraint realism.

Using the mutli-start algorithm, 84% of optimal kinematics obtained with previous isolated boundary joints were found to overpass 3D joints limits. Therefore, we believed optimal results realism was enhanced by the shoulder range of motion constraints definition. Further, the model would probably beneficiate from similar constraints about joint velocity [[51](#_ENREF_50)]. Finally, no statistical differences were found in the number of objective function calls (p=0.65), the number of feasible solution in the optimal (p=0.61), or the performances values found between the optimisations with 2D or 3D joint limits (p=0.92). First, the 3D shoulder constraint is believed not to increase computational cost of the optimisation. Second, the constraint implementation may not change a lot the optimal solutions, however its use should be recommended to improve realism of the solutions.

**REFERENCES**

51. Yunong Z, Jun W, Youshen X. A dual neural network for redundancy resolution of kinematically redundant manipulators subject to joint limits and joint velocity limits. Neural Networks, IEEE Transactions on. 2003;14(3):658-67. doi: 10.1109/tnn.2003.810607.

52. Shimizu M, Kakuya H, Woo-Keun Y, Kitagaki K, Kosuge K. Analytical Inverse Kinematic Computation for 7-DOF Redundant Manipulators With Joint Limits and Its Application to Redundancy Resolution. Robotics, IEEE Transactions on. 2008;24(5):1131-42. doi: 10.1109/tro.2008.2003266.

53. Wang X. Three-dimensional kinematic analysis of influence of hand orientation and joint limits on the control of arm postures and movements. Biol Cybern. 1999;80(6):449-63. doi: 10.1007/s004220050538.

54. Maurel W, Thalmann D. Human shoulder modeling including scapulo-thoracic constraint and joint sinus cones. Comput Graph. 2000;24(2):203-18. doi: <http://dx.doi.org/10.1016/S0097-8493(99)00155-7>.

55. Wilhelms J, Gelder AV. Fast and Easy Reach-Cone Joint Limits. Journal of Graphics Tools. 2001;6(2):27-41. doi: 10.1080/10867651.2001.10487539.

56. Tolani D, Badler N, Gallier J. A kinematic model of the human arm using triangular bézier spline surfaces. Graphical Models. 2000.

57. Herda L, Urtasun R, Fua P, Hanson A. Automatic determination of shoulder joint limits using quaternion field boundaries. The International Journal of Robotics Research. 2003;22(6):419-36.

58. Haering D, Raison M, Begon M. Measurement and description of three-dimensional shoulder range of motion with degrees of freedom interactions. J Biomech Eng. 2014;136(8):084502. doi: 10.1115/1.4027665. PubMed PMID: 24828544.

59. Tonneau S, Pettré J, Multon F. Using task efficiency contact configurations to animate creatures in arbitrary environments. Computer & Graphics. 2014;45:40-50.

60. Moya S, editor Accurate and computationnally faste joint range of motion parametrization. XIV International Symposium on Computer Simulation in Biomechanics; 2013; Natal, Brasil.

61. Caplan J, Julien TP, Michelson J, Neviaser RJ. Multidirectional instability of the shoulder in elite female gymnasts. American Journal of Orthopedics. 2007;36(12):660.

62. Kirby RL, Simms FC, Symington VJ, Garner JB. Flexibility and musculoskeletal symptomatology in female gymnasts and age-matched controls. Am J Sports Med. 1981;9(3):160-4. doi: 10.1177/036354658100900306.

63. McLaren K, Byrd E, Herzog M, Polikandriotis JA, Willimon SC. Impact shoulder angles correlate with impact wrist angles in standing back handspring in preadolescent and adolescent female gymnasts Int J Sports Phys Ther. 2015;10(3):341-6. Epub 2015/06/16. PubMed PMID: 26075149; PubMed Central PMCID: PMCPMC4458921.

64. Sheets AL, Hubbard M. Influence of optimization constraints in uneven parallel bar dismount swing simulations. Journal of Biomechanics. 2009;42(11):1685-91. doi: <http://dx.doi.org/10.1016/j.jbiomech.2009.04.014>.
