# Supplementary figures and images for: Identification of the contribution of contact and aerial biomechanical parameters in acrobatic performance
Source: PLoS One. 2017 Apr 19;12(4):e0172083. doi: 10.1371/journal.pone.0172083 (PMC5396868; doi:10.1371/journal.pone.0172083)

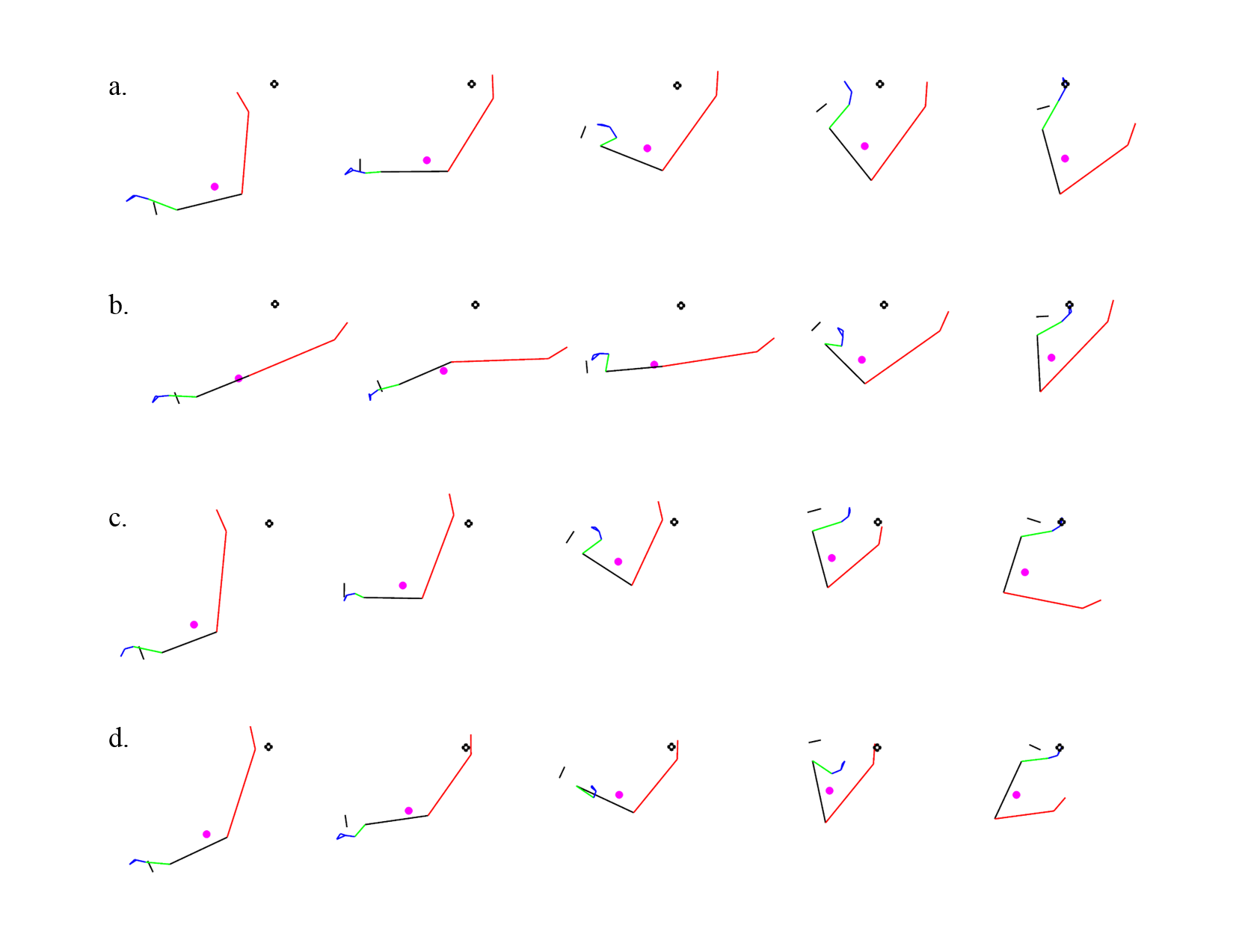

Supplement: S1 Fig — Novice recorded(a.), novice optimal (b.), advanced recorded (c.), and advanced optimal performances (d.). Smaller lengths in the (y,z) plane means the segments are more abducted towards x axis. (TIF) [file pone.0172083.s002.tif]

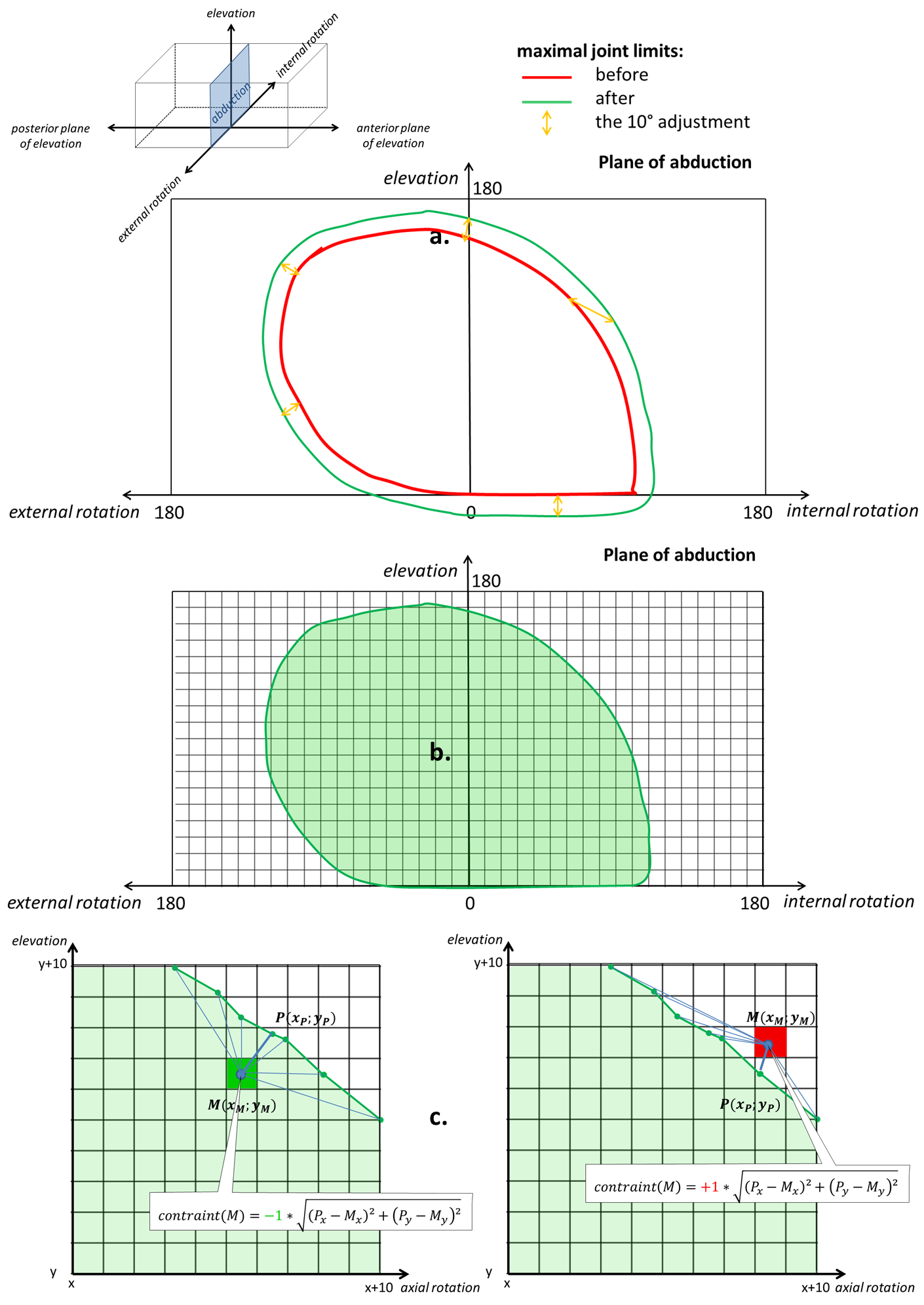

Supplement: S2 Fig — (a) joint limit definition and adjustment inside boundaries of a complete revolute joint, (b) discretization of the entire space and set the relative position to joint limits, (c) test of shoulder joint constraint for realistic (left) and unrealistic (right) poses. (TIFF) [file pone.0172083.s003.tiff]
